# Supplementary material for: Metals and Trace Elements in Calcified Valves in Patients with Acquired Severe Aortic Valve Stenosis: Is There a Connection with the Degeneration Process?
Source: J Pers Med. 2023 Feb 13;13(2):320. doi: 10.3390/jpm13020320 (PMC9967375; doi:10.3390/jpm13020320)
Supplement: Supplementary file 1 [file jpm-13-00320-s001.zip › Table S1.docx]

**Table S1.** Spearman-rank correlation between metals and trace elements in calcified aortic valve samples

|  | **Mg** | **P** | **Ca** | **S** | **Cr** | **Co** | **Cu** | **Zn** | **Se** | **Sr** | **Cd** | **Ba** | **Pb** | **Mo** | **Sn** |
| --- | --- | --- | --- | --- | --- | --- | --- | --- | --- | --- | --- | --- | --- | --- | --- |
| **Mg** |  | 0.57 | 0.58 | -0.56 | -0.02 | -0.14 | -0.48 | 0.48 | -0.63 | 0.46 | -0.12 | 0.33 | 0.24 | -0.09 | 0.21 |
| **P** | 0.57 |  | 0.99 | -0.77 | -0.24 | -0.49 | -0.55 | 0.41 | -0.49 | 0.54 | -0.51 | 0.23 | 0.09 | -0.10 | 0.01 |
| **Ca** | 0.58 | 0.99 |  | -0.80 | -0.24 | -0.50 | -0.60 | 0.43 | -0.52 | 0.54 | -0.50 | 0.23 | 0.10 | -0.10 | -0.01 |
| **S** | -0.56 | -0.77 | -0.80 |  | 0.26 | 0.39 | 0.60 | -0.41 | 0.76 | -0.42 | 0.39 | -0.12 | -0.21 | -0.08 | 0.04 |
| **Cr** | -0.02 | -0.24 | -0.24 | 0.26 |  | 0.37 | 0.10 | -0.05 | 0.02 | 0.12 | 0.02 | 0.10 | 0.17 | 0.07 | 0.26 |
| **Co** | -0.14 | -0.49 | -0.50 | 0.39 | 0.37 |  | 0.41 | -0.35 | 0.27 | 0.02 | 0.36 | 0.06 | 0.07 | 0.06 | 0.08 |
| **Cu** | -0.48 | -0.55 | -0.60 | 0.60 | 0.10 | 0.41 |  | -0.49 | 0.60 | -0.29 | 0.24 | -0.15 | -0.17 | 0.24 | -0.04 |
| **Zn** | 0.48 | 0.41 | 0.43 | -0.41 | -0.05 | -0.35 | -0.49 |  | -0.33 | 0.35 | -0.23 | 0.31 | 0.37 | 0.04 | 0.14 |
| **Se** | -0.63 | -0.49 | -0.52 | 0.76 | 0.02 | 0.27 | 0.60 | -0.33 |  | -0.30 | 0.09 | -0.05 | -0.29 | -0.04 | 0.12 |
| **Sr** | 0.46 | 0.54 | 0.54 | -0.42 | 0.12 | 0.02 | -0.29 | 0.35 | -0.30 |  | -0.20 | 0.44 | 0.18 | -0.07 | 0.25 |
| **Cd** | -0.12 | -0.51 | -0.50 | 0.39 | 0.02 | 0.36 | 0.24 | -0.23 | 0.09 | -0.20 |  | -0.03 | 0.04 | -0.04 | -0.20 |
| **Ba** | 0.33 | 0.23 | 0.23 | -0.12 | 0.10 | 0.06 | -0.15 | 0.31 | -0.05 | 0.44 | -0.03 |  | 0.33 | -0.22 | 0.00 |
| **Pb** | 0.24 | 0.09 | 0.10 | -0.21 | 0.17 | 0.07 | -0.17 | 0.37 | -0.29 | 0.18 | 0.04 | 0.33 |  | 0.19 | -0.32 |
| **Mo** | -0.09 | -0.10 | -0.10 | -0.08 | 0.07 | 0.06 | 0.24 | 0.04 | -0.04 | -0.07 | -0.04 | -0.22 | 0.19 |  | -0.04 |
| **Sn** | 0.21 | 0.01 | -0.01 | 0.04 | 0.26 | 0.08 | -0.04 | 0.14 | 0.12 | 0.25 | -0.20 | 0.00 | -0.32 | -0.04 |  |
