# Supplementary material for: Metals and Trace Elements in Calcified Valves in Patients with Acquired Severe Aortic Valve Stenosis: Is There a Connection with the Degeneration Process?
Source: J Pers Med. 2023 Feb 13;13(2):320. doi: 10.3390/jpm13020320 (PMC9967375; doi:10.3390/jpm13020320)
Supplement: Supplementary file 1 [file jpm-13-00320-s001.zip › Table S2.docx]

**Table S2.** Spearman-rank correlation between metals and trace elements in aortic valve samples from controls

|  | **Mg** | **P** | **Ca** | **S** | **Cr** | **Co** | **Cu** | **Zn** | **Se** | **Sr** | **Cd** | **Ba** | **Pb** | **Mo** | **Sn** |
| --- | --- | --- | --- | --- | --- | --- | --- | --- | --- | --- | --- | --- | --- | --- | --- |
| **Mg** |  | 0.94 | 0.71 | 0.54 | 0.25 | 0.43 | 0.47 | 0.79 | 0.25 | 0.63 | 0.39 | 0.61 | 0.69 | 0.48 | 0.15 |
| **P** | 0.94 |  | 0.84 | 0.37 | 0.23 | 0.43 | 0.32 | 0.68 | 0.08 | 0.74 | 0.51 | 0.68 | 0.77 | 0.39 | 0.17 |
| **Ca** | 0.71 | 0.84 |  | -0.01 | 0.19 | 0.38 | -0.04 | 0.44 | -0.27 | 0.90 | 0.41 | 0.73 | 0.78 | 0.08 | 0.14 |
| **S** | 0.54 | 0.37 | -0.01 |  | 0.01 | 0.17 | 0.81 | 0.62 | 0.77 | -0.11 | 0.09 | 0.01 | 0.12 | 0.44 | 0.19 |
| **Cr** | 0.25 | 0.23 | 0.19 | 0.01 |  | 0.33 | -0.02 | 0.32 | 0.17 | 0.26 | 0.23 | 0.28 | 0.20 | 0.06 | 0.00 |
| **Co** | 0.43 | 0.43 | 0.38 | 0.17 | 0.33 |  | 0.15 | 0.29 | 0.10 | 0.45 | 0.22 | 0.53 | 0.48 | 0.15 | 0.33 |
| **Cu** | 0.47 | 0.32 | -0.04 | 0.81 | -0.02 | 0.15 |  | 0.63 | 0.63 | -0.15 | 0.21 | 0.00 | 0.01 | 0.64 | 0.10 |
| **Zn** | 0.79 | 0.68 | 0.44 | 0.62 | 0.32 | 0.29 | 0.63 |  | 0.50 | 0.34 | 0.37 | 0.36 | 0.49 | 0.61 | 0.20 |
| **Se** | 0.25 | 0.08 | -0.27 | 0.77 | 0.17 | 0.10 | 0.63 | 0.50 |  | -0.26 | 0.16 | -0.17 | -0.10 | 0.52 | 0.13 |
| **Sr** | 0.63 | 0.74 | 0.90 | -0.11 | 0.26 | 0.45 | -0.15 | 0.34 | -0.26 |  | 0.33 | 0.82 | 0.77 | 0.05 | 0.00 |
| **Cd** | 0.39 | 0.51 | 0.41 | 0.09 | 0.23 | 0.22 | 0.21 | 0.37 | 0.16 | 0.33 |  | 0.25 | 0.48 | 0.45 | -0.11 |
| **Ba** | 0.61 | 0.68 | 0.73 | 0.01 | 0.28 | 0.53 | 0.00 | 0.36 | -0.17 | 0.82 | 0.25 |  | 0.69 | 0.02 | 0.16 |
| **Pb** | 0.69 | 0.77 | 0.78 | 0.12 | 0.20 | 0.48 | 0.01 | 0.49 | -0.10 | 0.77 | 0.48 | 0.69 |  | 0.12 | 0.14 |
| **Mo** | 0.48 | 0.39 | 0.08 | 0.44 | 0.06 | 0.15 | 0.64 | 0.61 | 0.52 | 0.05 | 0.45 | 0.02 | 0.12 |  | -0.13 |
| **Sn** | 0.15 | 0.17 | 0.14 | 0.19 | 0.00 | 0.33 | 0.10 | 0.20 | 0.13 | 0.00 | -0.11 | 0.16 | 0.14 | -0.13 |  |
